# #BodyPositive? A critical exploration of the body positive movement within physical cultures taking an intersectionality approach

**DOI:** 10.3389/fspor.2022.908580

**Published:** 2022-10-10

**Authors:** Meridith Griffin, K. Alysse Bailey, Kimberly J. Lopez

**Affiliations:** ^1^Department of Health, Aging & Society, McMaster University, Hamilton, ON, Canada; ^2^Department of Recreation and Leisure Studies, University of Waterloo, Waterloo, ON, Canada

**Keywords:** fat activism, intersectionality, physical activity, body positivity, black feminism, bodies of difference

## Abstract

Feminist activists and critical sport scholars in the global north have advocated for more inclusive representation of bodies and more accessible physical cultures. Body positivity, a contentious movement and concept, has been taken up in various ways by different groups. Some scholars believe it holds power to liberate individuals from patriarchal, neoliberal, capitalist, and colonial ideologies of what constitutes a “good” body. On the contrary, critics assert this movement has been gentrified by white-centered politics. Intersectionality has a similar genealogy as body positivity, with a rich history in Black feminist thought but now considered by many as coopted and whitened. In this article, we trace the rich and divergent legacies of both movements and explore at the structural level how body positivity is represented within physical cultures on Instagram. We use a social-justice oriented intersectionality framework exploring #BodyPositivity and #BodyPositive across a total of 141 posts using reflexive thematic analysis. We organize our findings into four themes: 1) Disclosure-Privilege of Body-Related Journeys; 2) The Absent-Present; 3) Consuming Positivity; and 4) Disrupting Normative Body Positivity Posts. Overall, we found that only certain bodies (and transformations) were visible within the data: those of (now) lean, white, cis-gendered individuals, many of whom were engaged in bodybuilding, and who were sharing their bodily transformation. We observe a remarkable absence of BIPOC, 2S LGBTQAI+, fat/thick/thicc/curvy, older, gender-nonconforming, and/or disabled representations. We also note the myriad ways that body positivity has been commodified and packaged into a product or service for consumption. Lastly, we outline and celebrate the exceptions to this norm where a minority of posts align more closely with the original intentions of the body positivity movement. We conclude with our position on how to *do* intersectionality research, and call on researchers to honor Black feminist origins and rich social justice history in these movements.

## Introduction

Body positivity—the idea that all bodies are good bodies—is a social movement and concept that is contested. It holds different meanings to different people, living in and with different bodies. It also has contradictory uptake within the movement, fitness, and wellness industry, wherein there is an inherent paradox between the predominant focus on body performance, improvement, and transformation vs. the body positive message to accept the body regardless of appearance or function. The body positive movement originated from fat, Black, and queer activism in response to certain bodies being so rarely visible or held as valuable in discourses and visual media (e.g., fashion or physical cultures). However, scholars question whether the #BodyPositive movement preserves, rather than disrupts, the status quo of white, straight, cis, and thin embodiments.

Similar to the body positivity movement, intersectionality has its roots in Black feminist thought, including the work of nineteenth-century anti-slavery and women's rights activist Sojourner Truth and Maria Stewart (1), and late twentieth-century theorists like Angela Davis, Audre Lorde, and members of the Black lesbian Combahee River Collective. The term was officially coined by Black feminist legal scholar Kimberlé Crenshaw in 1989 as a theory and analytic tool (2). Sociologist Patricia Hill Collins (3) followed by introducing the idea of the “matrix of domination” to describe the social organization of Black women's lives “in which intersecting oppressions originate, develop, and are contained” (p. 228) to highlight the importance of recognizing the complexity of intersecting structural dimensions of lived oppression, including body-related prejudice (gender, race, body size, etc.).

Intersectionality also has contentious uptake, as some Black scholars stress that intersectionality has been coopted. One example is the erasure of the history of intersectionality within intersectionality projects and write-ups, which Sirma Bilge (4) describes as part of a trend toward “depoliticizing intersectionality” (p. 405). This whitens and eliminates its function as an instrument for political change. Thus, intersectionality in research must recognize its rich history, and orient to interrogating the dynamics of power and oppression that are characterized by a reckoning with its founding purpose within Black feminist activism and scholarship (5). Intersectionality involves the exploration of race, gender, disability, sexuality, class, age, and other social categories and the interrelationship with systems such as colonialism, neoliberalism, and white supremacy (to name a few) that co-produce fluctuating and interlocking relations of power and oppression (6, 7). As intersectionality continues to gain traction (e.g., within the context of sport and physical activity), concerns have been raised that the social justice intent behind the movement may be sidelined when it is used merely to manage or analyze large amounts of complex data. For this reason, some social justice researchers (1, 5) call for social justice to be at the center of any research claiming intersectionality. This requires a deep commitment to anti-racist and feminist scholarship (5).

This context forms the backdrop for our paper, where we employ a social justice-oriented intersectionality framework to interrogate how the original activist intention of the body positivity movement can be understood or observed today on Instagram, specifically within physical cultures. We explore Instagram because in recent years, body positivity has become popularized through this photo-based social networking site. The body positivity movement is believed to have surfaced on Instagram in 2012, aiming to confront the unrealistic expectations and unrepresentative portrayals of women in media and advertising (8). Today, a search on Instagram of the hashtag #BodyPositive reveals 17.8 million posts and 9.8 million posts for the hashtag #BodyPositivity (Instagram, March 2022). Thus, social media activity surrounding body positivity offers a fruitful site of analysis for understanding complex intersectional privileges and harms embedded in this online space.

In what follows, we provide theoretical background on the body positivity movement as Black fat activism. Then, we provide empirical findings on how body positivity has taken shape on social media and the effect that has had on people's sense of embodiment and belonging. Subsequently, we present our analysis of a snapshot of body positivity Instagram posts, outlining how they privilege the disclosure of only certain body-related journeys, through a consumerist model of body positivity, in juxtaposition to a minority of posts that disrupt and resist these normative body positivity posts. Lastly, we explain how these insights and general approach can advance how to *do* intersectionality research in the sociology of sport.

### Body positivity: A movement for racial and fat justice

Although body positivity is intended to challenge body-related oppressions, such as exclusivity within physical cultures, fitness industries and popular culture have appropriated and commodified the body positive movement (9, 10) and have excluded older people, people from diverse races, individuals with physical disabilities, and gender non-conforming people (11, 12). Critics (who identify as body positivity activists) have lamented that the dominant norm for the “positive” body is still a young, white, lean, able-bodied, cis woman, and even though the movement often speaks about diversity and claims of intersectionality, it does not often show it (13). Sonia Renee Taylor, a Black and body positive activist, argues that if the movement is only positive for some bodies, it is not a body positive movement (14). Black Lives Matter activist Shackelford (15) refers to the coopted version of body positivity an example of “white feminism” within which the goal is not to challenge the systems that oppress all women—patriarchy, capitalism, imperialism, racism—but to succeed within them in ways that center white women. Cooper (16) identifies the current state of the body positivity movement as the product of gentrification, given its use has been appropriated, its origins erased or distorted, and a new white-centric version is sold back to the community to uphold white supremacy. On the contrary, some scholars still grasp on to hope that these types of movements can provide a haven for self-expression and individuality (17, 18) and that their basic original principles may well hold promise when it comes to promoting inclusion.

Body positivity was rooted in Black fat activism to resist the rise of anti-fat discourse in North America (19), and to refuse mainstream white thin appearance-focused representations that (continue to) discriminate against Black bodies. In general, fat activism is an unapologetic embrace and acceptance of fatness as a political identity and culture, seeking to challenge unjust stigmas and discrimination against people who are unjustly positioned as less worthy based solely on body size (16). Strings (20) traces the racial origin of the fear of fat, outlining how the contemporary ideal of slenderness both is racialized and racist, where fatphobia is not about health, but is instead a means to validate (whilst concealing) racial prejudice. Indeed, fat oppression can be traced through entangled systems of power and deeply rooted histories—many of which work to racialize fat, creating constructions and ideologies of fatness that are morally laden with stigma and judgment (21). Historically, Black bodies have endured problematic representation since, for example, the displaying of Saartjie Baartmen, the “Hottentot Venus.” Baartmen was a Black enslaved woman of size who was put on display in the early 1800s for white people to gawk at and touch for their own amusement (20). Barbara Christian, an American Black feminist critic, claims that, “the enslaved African woman became the basis for the definition of our society's Other” [(22), p. 160]. Collins (3) points out that maintaining and controlling images of Black women as the Other provides ideological justification for race, gender, and class oppression—and objectification is central to this process of oppositional difference. Importantly, even as the initial conditions that foster controlling images become less visible, such images prove to be enduring—because they work to subjugate, to marginalize, and to maintain intersecting oppressions. In today's general popular culture, the representation of Black bodies is either hyper-visible (i.e., via stereotypes) or invisible [i.e., erased; (23)]. For example, it is consistently found that fitness and sport magazines are dominated by white, young, tall, thin, seemingly affluent, happy, and able-bodied representations (24), and Black, fat, aging, and disabled bodies are nearly completely absent (25). In response to the often-problematic representation of bodies in media, queer theorists, disability scholars, and sociologists of the body have echoed calls from the body positivity movement to recognize the vast and valuable heterogeneity of the human bodily form (26). In response, many groups have advocated for more inclusive representation of diverse bodies and more inclusive and accessible physical cultures and spaces.

In early days of fat activism (1970's), a distinction emerged between radical and mainstream fat activism (27). Radical fat activists saw fat liberation as linked to other struggles of oppression, and mainstream fat activism often shut out the voices of people of color. The result was that this important movement, created to help marginalized folx, was experienced by some as marginalizing. As the fat rights movement grew (in the 1980's and 90's), the term “body positivity” was not yet being used—but enthusiasm for fat liberation was beginning to spread. Activists were drawing attention to fatphobic advertising, the damaging diet industry, and advocating for all to love their bodies. With the rise of the Internet, and social media in particular, the new millennium saw this movement spread online and body positivity became a social media buzz phrase influenced by capitalism. The distinction between radical and mainstream body positivity persists, but where early fat activism had not always made space for fat Black and brown people, Black and brown fat people were more able to carve out their own spaces online. Intersectional influencers who were dealing with oppression in more than one area were often the most outspoken, with women of color and queer folx often leading the way. The shift from a grassroots radical movement on the streets, to a mainstream commercialized social media movement, marks the beginning of a shift in how body positivity was taken up, losing touch with its origins.

### Body positivity on social media: Existing research

Achieving health, well-being, and the ideal body are often portrayed as a choice, achievable through individual concerted effort (28–33). Anyone who appears to “fail” to adopt a healthy lifestyle becomes a “failed citizen,” and their inability to take personal responsibility for their health explains and even *justifies* their discrimination (21, 34). Furthermore, individuals who cannot achieve the supposed “ideal body” (i.e., white, thin, affluent, and able-bodied) may be and/or may feel unable to access wellness and physical cultures and spaces, thus missing out on the positive health and social outcomes associated with physical activity and sport (35). Indeed, many spaces earmarked for physical activity proliferate dominant notions of ableism, racism, fatphobia, cis-sexism, heterosexism, and heteronormativity, erasing bodies of difference or, at best, casting them as non-normative (36–38).

With the growing use of social media, body image and physical activity have become a primary research focus among images on social networking sites (e.g., Facebook, Twitter, and Instagram (39). The literature suggests that exposure to media cultivating beauty ideals impacts body image, eating behaviors, and self-esteem (40). Positive correlations have also been observed between social networking site usage and body image concern in young adults and adolescents, internalization of the thin ideal and body dissatisfaction, dieting in adolescents, disordered eating level in young adults, and low self-esteem in young adults (41). Linking this to physical activity, Tiggemann and Zaccardo (42) explored the trend of “fitspiration” (fitness inspiration). Widely utilized on the social networking site Instagram, the “fitspiration” hashtag tends to be associated with images of women, typically engaging in exercise or dressed in exercise gear, or healthy food. The general philosophy is one which emphasizes strength and empowerment, but as Tiggemann and Zaccardo (42) explain, the narrow range of bodies depicted (thin, toned, white, etc.), and the motivational language that tends to focus on appearance-related benefits and objectification of body parts within the #fitspiration trend has the potential to have (unintended) negative consequences on body image.

There is an emerging literature exploring the effects of viewing body positive social media posts, but currently the findings are equivocal [e.g., (8, 11, 43–46)]. For instance, Cohen et al. (11) found that body positivity posts in their sample depicted a broad range of body sizes and appearances with messaging about positive body image (and de-emphasis on appearance) and that viewing body positive posts was associated with improvements in young women's positive mood, body satisfaction, and body appreciation, relative to viewing thin-ideal and appearance-neutral posts. Using ecological momentary assessment, Stevens and Griffiths (45) found that university-aged students viewing body positivity content on social media (Instagram, Facebook, YouTube, and Snap Chat) led to them experiencing higher body satisfaction and improved emotional well-being. They concluded that there is preliminary evidence to suggest that encouraging social media users to follow body positivity social media accounts may be a useful way to protect and enhance users' body image.

Adding to this literature, Tiggemann et al. (46) found that the visual imagery of a body positivity Instagram post was a more potent contributor to body image than any accompanying text or caption. Images of average-sized women as opposed to thin women had a more positive effect on body image in their sample of young women. On the contrary, Vendemia et al. (47) found in their experimental study that women who were exposed to sexualized or digitally manipulated body positivity posts on social media led to increased objectification (of self and others). This finding suggests that when body positivity imagery is sexualized or digitally altered (e.g., photoshop or filters), the result on people's body image actually undercuts the intended aims of the movement. Similarly, Brathwaite and DeAndrea (48) found that body positivity posts on Instagram that contained self-promotion or products were viewed as less morally appropriate and less effective at promoting body appreciation and inclusivity. In our study, we build on this body of work by demonstrating how social media enactments of body positivity may not recognize or honor the Black fat and queer feminist origins of the movement, thereby privileging only normative body-related journeys through a white consumerist model of body positivity.

### Current study

Interestingly, very few publications have used an intersectionality framework or acknowledged the vibrant Black and queer activism that started the body positivity movement. We address these gaps by exploring the hashtags #BodyPositivity and #BodyPositive on Instagram using the lens of intersectionality, exploring how Black, queer, fat, and other bodies of difference are represented and mobilized in these spheres in (what was) a social justice and activist movement.

We are engaging with intersectionality by leaning into a social justice-oriented framework to understand the twists and turns of the body positivity movement, with a close examination (a snapshot) of how that has materialized online in Instagram, with respect to physical activity or movement. We do this by not merely exploring micro-level “differences” or “identities,” but the macro and structural-level entanglements that may explain operations of power in our data. Thus, we take an anti-racist, -ageist, -ableist, and -sexist feminist lens to explore from a critical vantage point: (1) how body positivity manifests and is leveraged by certain/particular bodies in active and embodied spaces (physical cultures); (2) how body positivity as it pertains to, or is associated with, movement subverts or reproduces oppressive body-related logics; and (3) how the original activist intention of the body positivity movement can be understood or observed today. We also uphold a conscious awareness of intersectionality's origins and commit to a critical application of this framework to understand how both intersectionality and body positivity, as Black activist movements, have been coopted, diverted, reshaped, and whitened.

Since we are engaging in intersectionality research, and to be consistent with calls for critical reflexivity when *doing* intersectionality (5, 49) we feel it is important to delve into some of our own positionality to the topic. Doing so provides an opportunity for us to disclose our research motivations (what brought us to ask these questions), but also to make evident that our positioning situates our chosen theoretical, methodological, and analytical processes—most importantly, shaping how we both collected and interpreted our data, and how we here seek to communicate our findings (50). We have varying degrees of relationship to non-normative embodiment and take a critical feminist orientation within the study of leisure, recreation, kinesiology, and social psychology. All of us are cis women, two of us are white settlers (one with Irish descent and the other with English, Irish, and Scottish ancestry), and one is of colonized Filipinx ancestry. We have varying relationships to queer, thick/thicc, and Mad identities, and complex histories of eating/dieting/exercising/sport-related struggles. Lastly, we all reside on Turtle Island (also known as Canada), in Southern Ontario. While this brief positionality statement does not fully capture the nuance and fluidity (50) of our relationship to our research and the complexity of intersectionality, we believe it is important to provide the reader a sense of our subjectivities.

### Data collection and procedures

We collected data from Instagram over a 5-week period in October-November 2021, a time period selected to provide a breadth of representation while limiting the quantity of data (so as not to be overwhelmed). We pulled posts from a Tuesday, Wednesday, Friday, Saturday, and Sunday, on separate weeks across different times of the day (morning, afternoon, and evening), seeking to control for and/or include weekend vs. weekday patterns and time of day idiosyncrasies. We sought to avoid the inclusion of holidays and special occasions (in our case, Yom Kippur, Thanksgiving, and Halloween), as we were trying to capture everyday patterns rather than be influenced by special occasions. It is important to note that data were collected during the ongoing global COVID-19 pandemic, which may have shaped online practices, habits, and activities—including social media use. Only publicly available posts were included, where an individual's profile and posts can be seen by anyone, on or off Instagram, regardless of whether they have an Instagram account. Private posts, where only approved followers can see what is shared, were not included in our sample. We chose Instagram because it has catapulted to being one of the most popular social networking sites and photo and video-sharing platforms. Currently, it has two billion+ active users, 25 million+ business profiles, and 500,000+ active influencers, who are approximately split according to a gender binary (48.4% female, 51.8% male, no data on other genders), are of all ages (largest age group is 25–34), and racially diverse (51, 52). Furthermore, Instagram is home to both feminist-related discourse and activism but also concomitant with a problematic rise in misogyny (53) and other neoliberalized, colonized, and gendered imperatives about “worthy” bodies (54).

We began by searching for the hashtags #BodyPositivity and #BodyPositive, pulling 50 of the most recent posts of each on each day. After some deliberation and exploration, we selected to analyze “recent posts' rather than “top posts” (which are the most popular posts tagged with the chosen hashtag) because the “top posts” appeared to be driven by influencers and be more normative (more white, thin, able-bodied, young, etc.), while recent posts captured representations by anyone that chose to use the hashtag^1^. Our search resulted in 500 posts total, which we then searched through and coded for physical activity-related information and removed posts that were not written in English. We observed that our data, even the English-only posts, were internationally represented (e.g., Russia, United Kingdom, United States, Greece, Italy, and Brazil), demonstrating considerable reach of this movement on a global-scale. Posts were broadly coded as containing “physical-activity” if: the image(s)/video(s) depicted any type of physical activity or physical activity setting (e.g., gym, outside on a hiking trail), or if fitness/athletic apparel were worn (loosely defined: leggings, sports bra, shorts, t-shirt, swimsuit); the caption mentioned movement, sport, or activity of any kind; or if hashtags and/or comments mentioned physical activity in any manner (e.g., #Fitness, #BodyBuilding, #Dance). This resulted in a total dataset of 141 posts for the purposes of the current study.

We stored screenshots of all the posts in a Word document for analysis. Since we were using public Instagram posts that had no reasonable expectation for privacy, our university ethics board did not require a review of our study nor did we require informed consent from Instagram users. That said, we recognize that definitions of public and private are complex, nuanced, and dynamic, and relying on a simple understanding of “publicly available” is not sufficient for social media research to be ethical (55). As such, in our analysis below, even though we are legally allowed to re-use this publicly available information, we deliberately chose not to reveal users' Instagram handles or reproduce any of the images^2^. The choice to describe rather than reproduce the visual material alongside textual material (captions, hashtags, and comments), was made in an effort to draw greater attention to the actions of the images, discourses, and affects than to the individuals themselves, since this better aligns with our intersectionality approach to our work. We invite reflection on the ways that we (researchers and readers) may be implicated in the uptake and spread of problematic imagery and values. While this does not comprehensively address all ethical and privacy concerns (57), we also chose to present our data anonymously out of respect for individuals who may be grappling with body issues and body-related politics.

### Data analysis

We approached analysis using Braun and Clarke's (58) reflexive thematic analysis to understand the data. In recent publications, Braun and Clarke (59) argue for new, theoretically rich, mashups of reflexive thematic analysis that take a critical approach to interpretation. We understand Instagram media as mixed media—as informing analysis across textual, audio, and visual data. From this view, Instagram might be thought of as behaving like other media in engaging all the senses to different degrees and in relying on text and other signs (e.g., sounds, what is absent) to generate meaning. The coming together of text and image creates a multimodal text (60) where we searched for meaning in image captions, hashtags, images and videos, as well as in interactions among images, videos, and texts.

We began by generating a reflexive thematic analysis through identifying and categorizing the image and textual content of the posts gathered. The authors familiarized themselves with the data by looking over the images and videos and reading the captions and comments (61). They then proceeded with an initial inductive analysis of the posts that described image content and composition, hashtags used, number of comments and likes, and other general patterns operating across posts (i.e., use of images, videos, captions, and comments). From there, posts were sorted into preliminary themes based on the coded commonalities and differences observed across text and images. The researchers completed this stage of analysis through regular meetings where they discussed possible theme names and content. An integral aspect of our theoretical approach to this project was taking up a social justice-oriented intersectionality lens with respect to these data. We explored how political and economical structures materialized within the body positivity and physical activity spheres in ways that either expand or limit possibilities for bodies of difference.

## Findings

### Disclosure-privilege of body-related journeys

Across the posts, we observed a tendency for people to disclose intimate and personal body-related stories as it related to their body positivity journeys. These disclosures included content about body transformation mostly regarding losing weight, gaining muscle or lean muscle mass, engaging in more physical activity, and learning to be kinder to their bodies. These posts comprised nearly one-half of the total dataset and included common hashtags such as #WeightLossJourney #WellnessJourney #WeightLossTransformation #FitnessJourney #BodyTransformation #MyTransformation #SummerBody.

For us, this raised the question of why people were compelled to share intimate/ personal journeys about their bodies. We posited that this could be linked to accountability, wherein individuals were seeking to be transparent about where they started from and/or how hard they have worked (or need to keep working) in order to maintain momentum. Alternatively, in sharing these journeys, it could be interpreted as a claim to increasing body status and thus social capital. We also observed a significant trend within these data: that it was mostly white, thin/lean, bodybuilding (seemingly cis and able-bodied), men and women (within a gender binary) who were freely discussing these stories and displaying images of their bodies within this Instagram hashtag. This provoked the question: who has *access* or *permission* to share their personal body-related stories with more or less scrutiny?

Access to take up space, whether it is physical space in a room or virtual space within an online platform, is rooted in political, racialized, and gendered intersections of power, privilege, and oppression. Whose voices and representations are amplified (determined “worthy” of a click, or view, or a like) on Instagram is shaped by racist, ableist, ageist, sexist (and so on) structures that are embedded within the Global North (16, 21, 26). Accessibility to online spaces is significantly affected by gatekeeping, wherein so-called non-normative bodies (or bodies that do not align with the dominant ideal) are silenced by a set of unwritten but unambiguous rules about whose bodies “deserve” positivity (9, 12). Alternative creators (or representations) are thus silenced through these dominant and overlapping social forces that privilege some representations over others (13). We posit that although others within broader society may yearn for connection and validation through their Instagram posts, this comes with considerable risk—expressing vulnerability (through sharing images, personal stories, or experiences that are not typically celebrated) often results in trolling and/or bullying, bringing with it considerable concomitant mental health and safety implications. Within the posts in our study, white muscular men and thin/lean/toned white women who powerlift freely posted about their body-related journeys of fitness and body acceptance. For example, posts consisted of white men flexing their back toward the camera, women and men sharing videos of recommended exercise sequences, as well as before and after images of weight loss or muscle gain.

In one post, a white hyper-muscular male posted a before and after image of “6 months solid work with [tags another user] …,” alongside one comment that said, “what a transformation in every aspect my man!!” The other comment on this post was a business proposition. In another post, a white lean woman is standing in a semi-squat position on her bathtub flexing her biceps and smiling. The caption read:

Forgot to post how strong I'm feeling!!! I have rough days and today is NOT one of those days! Almost done with another program and after finishing each program makes me so proud of myself for following through! I need to continue to follow through with things I would say it can be my downfall. But today we celebrate 
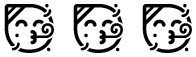
 I'm buying new clothes for this new body! (NOT WORKOUT CLOTHES EITHER).

Similarly, one user posted a before and after photo of her weight loss journey. The caption said:

It's crazy how much I've changed in the last 2 years. The first photo with the gray cardigan I was weighing 175lbs and the second is my now and weighing at 130lbs. My wellness journey has not been easy but this is just beginning and I'm looking forward to my future. I'm giving it my all!! Let's get it. Don't ever give up!! Push thru it and your body, soul and mind will thank you.

This post was also paired with the hashtags #HealthyLifeStyle #WorkThruYourPain #SelfLove #MindBodyAndSoul #LetsGetIt #FitnessJourney #ILoveMe #BodyPositivity.

Another post was of a white woman and her young toddler. The caption read, “Not the best sleep night for this congested little man (or me) but he's in good spirits and this mama needs some movement 
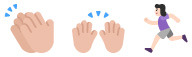
 HAPPY FRIYAY!! 
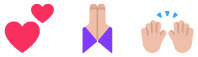
”, and #BodyPositivity was paired with other hashtags including: #HealthyLiving #FitAfterBaby #FitnessMotivation #FitMommas #FitMom #Inspiration #FitMomsOfInstagram #PostPartumBody #FirstTimeMom.

One user posted an image of herself kneeling on the beach with short shorts and a sweater. The caption read:

number one BIGGEST mistake when looking for help in your journey [is] copying what someone else is doing “oh, sally ate oats for breakfast, chicken and broccoli for lunch and dinner and hard boiled eggs for a snack and worked out 6 days a week and lost 15lbs, so I should do the same” and then you do and hate everything about food and working out and still don't lose 15lbs…..
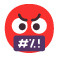

The post was accompanied by the hashtags: #LifestyleCoach #MindSetShift #FitnessGoals #LifeGoals #HealthyLifestyle #BodyPositivity #LiveALifeYouLove.

In summary, most users who dare to disclose these intimate body-related journeys and struggles are white, seemingly cis, straight, young, abled-bodied men and women who have very strictly (and successfully) abided by body disciplining instructions and narrow ideologies of what a body can and should be. These findings indicate a considerable departure from the original intention behind body positivity as a movement to disrupt white and Euro-centric logic about what constitutes a “good” body. By corollary bodies of difference either dare less to disclose, are fearful of social or other repercussions, or they actively decide not to align themselves with a movement that no longer represents, supports, or serves them.

### The absent-present

It may be that the association of body positivity with certain transformative (and mainstream) representations—visible within the previous themes—may be distasteful to more activist-oriented individuals who are keen to disrupt such associations. Certainly, in these data, particularly within the disclosure of body-related journeys, we observe a conspicuous absence of explicit images of Black, Indigenous, Person of Color, fat, thick/thicc, plus sized people, disabled people, 2SLGBTQAI+ people, aging bodies, or overall representation of non-normative bodies, and a lack of diversity in types of physical activity modalities. Our metrics indicate that one third of the total posts are of bodybuilding or powerlifting, less than one fifth include BIPOC representation, only three posts of 2S LGBTQAI+ people (all of whom were gay men), less than one sixth of the posts included body size diversity, only seven with reference to aging bodies (only two with explicit visual representation), and only two posts containing any mention of disability.

This is noteworthy, considering body positivity as a movement began because of these very embodiments and body-related politics. Upon further investigation, we noticed that some racialized women would post within this #BodyPositivity sphere, but not with images of their bodies. For example, one post was of a zoomed-in photograph of a smart watch. The caption read:

Your girl, Sam, got all her steps in on this Sunday. I must say I'm very proud of myself 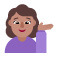. It's been a struggle recently. But today I got it in, all while enjoying myself with my Sonshine at the zoo!! In the words of Ice Cube… today was a good day 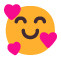

When looking through her accompanying hashtags (e.g., #BlackPodcast #BlackGirlMagic #BlackGirlsDoYoga #BlackGirlsWorkout #BodyPositivity #PlusSize) we realized the user was a Black woman. Another similar post had an image of a pink background with text reading, “Love yourself enough to: feed your body with real food, find time to exercise, give yourself time to rest, cut off bad habits.” The caption said, “Self-love is not just an abstract concept. Take action today to nourish your body and soul.” The post included the hashtag #BlackWomenHealth and the user account was a non-profit wellness lounge tailored for BIPOC women to help mitigate health disparities. This, again, is another example with no explicit visual display or representation of BIPOC. It is difficult to speculate as to the unarticulated motivations of post creators such as this one, but we suggest that all such choices are inevitably infused with body politics, and specifically the systematic oppression of Black female bodies. Perhaps choosing to not represent their bodies explicitly is tied to a structural tendency for BIPOC women to be either invisible OR hyper-visible and represented in stereotyped and commodified ways (23).

### Consuming positivity

Another significant trend within our data was that more than one quarter of the posts (38/141) included either a direct URL link to a product or service or a hashtag linking to an obvious product or service. For many, this included links to workout clothing (e.g., primarily sports bras, leggings, and swimwear).

One example includes an image of a young woman, blonde hair in braids and sitting on what appears to be a park picnic bench, measuring out some protein powder into a small scoop. Looking at the scoop and not the camera, she is wearing black leggings and a red sports bra-like top. The caption reads: “RED-y for a workout? [tagged another user] we are with you babe! Wearing the Kiwi Cross Back top in red 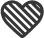 Shop online now 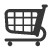 [with URL embedded]. The hashtags continue the sales pitch, reading: #KissMyPeachSwimwear #Bikini #BikiniBody #BikiniInspo #BikiniGirl #BikiniBabes #BodyPositivity #EveryBodyIsABikiniBody #Bikiniszn.

The idea, as we interpret it, is for the audience to purchase and thus emulate either the body depicted in the image itself OR the apparent empowerment being exemplified within the post/poster's body. Relatedly, protein powder and other nutritional supplements also featured heavily, particularly in the posts depicting bodybuilders, wherein #BodyPositivity would be mentioned in the same hashtag list as #PhysiqueFreak, #FitnessMotivation, and the like.

Also present were clinics selling aesthetic services, which mention #BodyPositivity in the same breath as various surgical and non-surgical cosmetic procedures focused on reshaping the ‘problem' or non-normative body (e.g., #BodySculpting #BodyContouring #SkinTighteningTreatment #TummyTuckSurgery #BrazilianButtLift #Liposuction). Such posts are thinly veiled references to the idea that we all have problematic bodies (though, of course, some more than others) that require investment in the efforts of professionals to help to contain, control, and even create the supposed ideal body (which, once achieved/purchased, one could presumably be positive about).

Perhaps unsurprisingly, personal trainers and small gym owners featured heavily here. Indeed, the “count” could well be higher in that we did not include general references to personal training (e.g., where it said #PersonalTrainer, or #GymName, but it was not clear whether the post was driven by the trainer or the gym, or just cited by the user of the service). We also did not include (in this count) the several instances where workout descriptions and/or demonstrations were posted, unless the reader was also encouraged to sign up for a larger “program” or service offered by the poster/trainer.

One example of this type of post includes an image of a dark-haired, tanned, toned woman who has one foot up on a weight bench, and is performing a bicep curl. The accompanying text is rife with affirmations:

Believe in yourself and all that you are
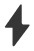

Know that there is something inside you that is GREATER than any obstacle!!

Train with a fire inside you and achieve your goals 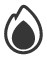

It only takes one spark to light a fire inside you, what's your spark?

Click in the link in our BIO to come try 7 days of Unlimited classes.

The hashtags accompanying #BodyPositivity on this post underline the integral role of this particular gym in helping to shape a body worthy of being positive about: #CircuitTraining #FitnessJourney #FitnessGoals #LightYourFire #InstaFit #NotAGymButACommunity.

Other posts of this ilk used similar language and imagery, depicting an individual engaged in movement—usually strength training—or flexing their seemingly “hard-earned” muscles in a mirror selfie. Bodybuilders were frequently at the forefront in these posts, whereby personal trainer(s) demonstrated the product that their expertise could help to create, with an accompanying URL to purchase their training program. Here, #BodyTransformation was used more than once immediately alongside #BodyPositivity—a jarringly ironic juxtaposition.

Less aesthetically focused bodywork services were also present within the collected posts, with massage therapy being the predominant example. These practices were being marketed as a form of self-care, part of a larger body project that includes a responsibility to invest in your own wellness: #Massage #MassageTherapy #Spa #Relax #Wellness #Beauty #MassageTherapist #Health #Selfcare #Relaxation #Fitness #DeepTissueMassage #SportsMassage #Healing #Therapy #HealthyLifestyle.

The implication is that you must consume (and spend!) on these bodywork services in order to be positive about your body. The commodification of wellness is readily apparent in these data, broaching a wide range of products and services and perhaps best exemplified within the posts created by wellness coaches. Citing self-love via bodily practices (upon which they can advise!), these coaches used a range of hashtags and affirmational quotations to call the reader to action, “to nourish your body and soul.” This is particularly insidious because at first glance, it largely aligns with the original messages of the body positivity movement. For example, one such post features the text, “Healthy is an outfit that looks good on everybody,” alongside the caption: “There is no one healthy shape, clothing size, weight, or age – everyone's version of a healthy body is different and equally valid.” The accompanying image, however, is once again a toned/slim, white, blonde, and seemingly able-bodied cis-appearing woman looking downward and wearing a sports bra, weight-lifting gloves, and headphones around her neck.

Again, the message is that we can all be positive about our bodies, but doing so requires engagement in individualized and morally laden health practices including plenty of movement/activity, balanced nutrition, recommended amounts of sleep, etc. In so doing, we have the best chance of achieving happiness and are able (enlightened?) to “#LiveALifeYouLove.” Regardless of whether this is true (for some) or not, it is evident that what started out as a social movement pushing for inclusivity and body acceptance is now one characterized by an imperative to try to attain the ideal body through consumption and discipline (#Hustle). Most important to note here is that consumption of the material goods associated with feeling positive about one's body—clothing, nutrition, exercise, massage, etc. —is contingent on structural and systemic parameters like economics, time, and resources which are well-documented to be inequitably distributed on the basis of race, class, gender, ability, and the intersections thereof (28–30, 33).

### Disrupting normative body positivity posts

It is worth noting that there were some posts (albeit a minority) in our sample that functioned to resist or interrupt the coopted body positivity and physical activity movement on Instagram. These posts disrupt some of the normative content or even interrogate it. This includes difference affirming images, captions, and hashtags, as well as race, body size, age, and physical activity diversity. A common trend in these posts was a focus on movement being for enjoyment rather than changing/altering/disciplining the body.

These five posts helped disrupt the normative content in the #BodyPositivity sphere by providing alternative perspectives and imagery that may even have a provocative effect. For example, one post had a gray-scale image of an older adult white woman in a white bikini, standing with a proud pose, hands on her hips, stern facial expression and eyes looking into the camera. The caption said, “The female body was never supposed to be smooth, firm, and flawless. It was designed to create life, to host life, to feed life…” and the user is an “African dance and fitness studio” owner. Another post was of an image of a racially and body size diverse group of people laughing and posing in front of the mirror in a Zumba studio.

The third post was a video of a curvy racialized woman demonstrating a high knees running on the spot exercise with the caption, “I get very conscious about how bad I look while running or jumping others must be making fun of me by saying see one panda is jumping …I've overcome from body shaming 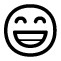 I love how it looks how huge it is, after all it's mine and we love each other 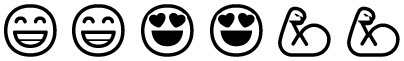
”, and #BodyPositivity was paired with fat-affirming hashtags #CurvyWoman and #CurvyAndFit.

Another post advertised how to exercise for joy rather than fear, guilt, or shame. A series of five images was posted. The first image was of a white thick/thicc young girl smiling at the camera. She had a tattoo on her right arm, a messy ponytail blowing in the wind, and was holding yellow dumbbells in both hands. The second-third images were of four young-middle aged women/gender ambivalent people with arms locked and laughing. The fourth image was zoomed in on a young (plus-sized) woman's face as she smiled at the camera. The last image was of a thick/thicc middle-aged to older-adult woman outside on the ground on a yoga mat stretching her back and thighs.

The last example in this theme is a post with the juxtaposition between two images: one of a white baby's naked legs and baby fat rolls and the other of a young adult white woman in a bathing suit revealing fat rolls and cellulite on her legs. The caption said, “To which we say, if we love the one on the left, then you can absolutely, positively love the one on the right too 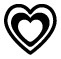
”. This post invites the viewer to question why fat is seen as adorable on babies but is approached with disdain when on adults (especially adult women).

## Conclusion

In conclusion, our study supports findings and assertions that the body positivity movement is multi-faceted and deeply divided (62). Overall, we found that within our sample of body positivity posts depicting or referencing physical activity, there was a predominant representation of (now) lean, white, cis-gender individuals, many of whom used bodybuilding as their primary physical activity modality. Along with this finding was a conspicuous absence of fat embodiments, BIPOC, disabled people, 2SLGBTQAI+ people, or aging bodies. With just a few exceptions, the Instagram posts we found linked #BodyPositivity to physical movement/exercise/healthy lifestyle to a very narrow visual field that was overwhelmingly white, visibly physically able, and demonstrating strength by appearance and function. In our initial pull of 500 posts there was a mixture of fashion and physical activity related posts. In our observation, the physical activity posts were particularly problematic in representation. In the context of fitness and movement motivation-type posts, the use of #BodyPositivity is used to reify status-quo, idealizations of white bodies, coopting the more inclusive Black body positivity movement. In some instances, it appeared that users somewhat haphazardly applied #BodyPositivity to their posts alongside, in some cases, dozens of other body/fitness related hashtags without acknowledging what the visual presence of whiteness and physical able-ness does in its occupation of space where the Black body positivity movement started.

With a few exceptions, what most of the posts we located fail to acknowledge, let alone refuse, are the structures that cause oppression of non-normative bodies. From an intersectionality lens, this is problematic because it works to make light of how the visual “in-club” or the gold-standard of fitness—the array of sculpted, light-skinned bodies—ignoring the sentiment of a movement meant to sit in critique of a system that oppresses bodies outside of this norm. An occupation of #BodyPositivity by the visual status quo, where previously absent, erases from where the movement began. In some cases, this re-territorialization of social media presence works toward elite capture or the promotion of capitalist ventures that privilege just a few (63–67) deepening the division between who is able to be represented as body positive and minoritized groups who started #BodyPositivity. In other ways it fails to address the tension between #BodyPositivity and potentially harmful practices (e.g., diet culture, disciplining the body) to attain specific body standards and how much more unrealistic it becomes for individuals who fit outside of the standard template to connect with #BodyPositivity as it has been claimed and reframed. The coopting of #BodyPositivity in this way changes the movement that once had a strong connection to Black activists and supported by other activists of color to that which is applicable to a few. #BodyPositivity instead of being a space for opening up an inclusion of all through allyship and celebration became, “reduced to an identity, not a movement” [(68), p. 6].

As a field, leisure studies has used inclusion as a guise for leisure being perceived as always “good” when it is marketed as “leisure for all” (57). Despite calls to adopt intersectionality more widely in leisure studies (69), efforts for inclusion consistently occlude the fact that leisure and leisure time is often systemically out of reach for individuals who are disabled, of color, old, fat, or queer [e.g., (70–73)]. While it has always been necessary to critique the exclusion or inclusion of individuals based on race, since recent and widespread awakenings to race it is more vital to describe what the intrusion of whiteness or exclusion of Blackness (and other communities of color) does to a movement based on optics, particularly while a greater proportion of people are becoming more sensitized to the damages that occur from these taken-for-granted relations of dominance. Based on hashtags, Instagram can give us a good sense of whether it is an intentional opening up to include folx outside the initial spurring of the cause, band wagoning, or other intentions to be seen through affiliations with #BodyPositivity (i.e., allyship) that is occurring. The Blackness of the #BodyPositivity movement simultaneously resisted dominations of white-body as the default beautiful-body while also making the Black body visible at multiple intersections (12, 69, 71). Like many other cultural spaces, including those of leisure (i.e., music, art, dance, fashion, celebration), we are seeing a familiar migration of whiteness into #BodyPositivity, what was once a Black-centered movement to celebrate all-shaped Black bodies. Given the historicities of the various movements we discuss, race is central to the analysis of intersectionality and body positivity given their origins in Black feminism. Our data appears to take up race in ways that “legitimizes” fitness through alignments with whiteness and slimness. We believe it is necessary to critique leisure studies as being a predominately white institution that has conventionally failed to critique race by making bodies raceless through their emphasis on disability and class. It is critical that movements within disability, queer, and fat studies (and more) recognize their entrenchment in white supremacy. For example, queer inclusion tends to re-center whiteness and preclude Black queer experiences [e.g., (74)]; disability studies has “whitewashed” disability [e.g., (75)]; and fat studies has a tradition of centring white experience irrespective of the racist origins of fatphobia and “obesity science” (20, 21).

In this instance, #BodyPositivity has become another arena of appropriation where privileges are experienced very differently between person to person. Even the now-desirable “curvy” or thick/thicc body was born out of the need to recognize, represent, and celebrate Black bodies that were too often made invisible or worthless because of sizeism (16) and racism (20). The mainstreaming of thickness/thiccness (76) in itself is a maneuver to coopt a movement that privileges and promotes white voluptuousness over Black thickness, and the accompanying practices for body augmentation needed to acquire the various iterations of body ideals. The incessant and historical erasure of Black fat activists' radical and global lens on oppression is an integral aspect of body positivity's gentrification. Both body positivity and intersectionality share a similar genealogy, and we must confront and resist the cooptation and whitening of both movements. We observed that most studies on #BodyPositivity (particularly about body image) did not recognize the rich Black, fat, and queer history of that movement. This glossing over (or blatant erasure) has the insidious effect of re-asserting white supremacy through operations of ignorance. When *doing* intersectionality research within sport, physical activity, and leisure, we call for researchers to honor this history by bringing it to the fore and by doing so, seek a social justice-oriented lens in their work on intersectionality.

Feminist organizing in digital spaces, such as Instagram, has the potential for activism, resistance, and visibility, but may also continue to be shaped by unequal power dynamics (62). Movements such as #MeToo (about sexual harassment) and #TimesUp (a campaign to end gender-based discrimination in the workplace), turned into “real world” activism. However, this is concomitant with problematic sexist, racist, classist, sanist (and other “isms”), that subvert the original intent of some movements or, the blurring of boundaries of various feminisms (and postfeminist sensibilities). As Banet-Weiser [(53), p. 11] points out, the “feminist” content that is likely to gain traction on Instagram is that which poses the least challenge to established heteronormative, patriarchal, gendered, racial and classed structures. It may be that Instagram, as a platform, constrains (and even suppresses) less palatable instances or frames for feminism, instead amplifying images and voices that align with (rather than deviate from, or protest against) the prevailing norm (77). However, given its reach and user demographic, how Instagram is used (and potentially subverted) by activists and other users alike warrants attention. With respect to the body positivity movement and its association with physical activity, this matters because whose bodies are visible/displayed as worthy of being positive, while doing or discussing physical activity, filters down into messages about who CAN do activity, how you should look while doing it, and what you should buy to get the most out of it. If “mainstream” body positivity continues to dominate the social media sphere, it is not a stretch to say that this is de-motivating at best, and discouragingly exclusive at worst for those not represented therein. Furthermore, the current body positivity's detachment from the original Black fat-centered radical movement functions to re-center whiteness and white supremacy, in potentially dangerous and insidious ways masked as “a good thing” (i.e., body positive). Thus, we echo other feminist scholars' [e.g., (1, 5)] call to re-center race in intersectionality research, which includes honoring the Black feminist and social justice history of the movement and interrogating the reproduction of white supremacy across social movements.

## Data availability statement

The raw data supporting the conclusions of this article will be made available by the authors, without undue reservation.

## Author contributions

MG conceptualized the original idea of the study and contributed to analysis and writing of the manuscript. KB assisted in conceptualizing the study, contributed to the study design, analysis, and writing of the manuscript. KL assisted with analysis and writing of the manuscript. All authors contributed to the article and approved the submitted version.

## Funding

This project was supported in part by funding from the Social Sciences and Humanities Research Council (#435-2021-0425).

## Conflict of interest

The authors declare that the research was conducted in the absence of any commercial or financial relationships that could be construed as a potential conflict of interest.

## Publisher's note

All claims expressed in this article are solely those of the authors and do not necessarily represent those of their affiliated organizations, or those of the publisher, the editors and the reviewers. Any product that may be evaluated in this article, or claim that may be made by its manufacturer, is not guaranteed or endorsed by the publisher.

## Footnotes

^1^This finding may well vary according to when Instagram is accessed and by whom, but the important thing to note is that our data captured current/recent posts on each day, and so does not necessarily speak to the posts that received the most audience engagement or reach but rather a mixture of influencers and everyday less popular users.

^2^This choice was made because there is no guarantee that even those who publicly share their information on the Internet would be supportive of it being used in research. As such, there is a potential mismatch between the expectations of the researcher(s) and “the researched” regarding the public/private distinction (56).
